# Determination of the Absolute Configurations of Chiral Drugs Using Chiroptical Spectroscopy

**DOI:** 10.3390/molecules21081056

**Published:** 2016-08-12

**Authors:** Prasad L. Polavarapu

**Affiliations:** Department of Chemistry, Vanderbilt University, Nashville, TN 37235, USA; Prasad.L.Polavarapu@Vanderbilt.edu; Tel.: +1-615-322-2836

**Keywords:** chiral, spectroscopy, drugs, electronic circular dichroism, optical rotation, vibrational circular dichroism, vibrational Raman optical activity, absolute configuration, conformation

## Abstract

Chiroptical spectroscopy has emerged as a promising tool for the determination of absolute configurations and predominant conformations of chiral molecules in academic laboratories. This promise has led to the adaption of chiroptical spectroscopic methods as valuable tools in chiral drug discovery research programs of the pharmaceutical industry. Most major pharmaceutical companies have invested in in-house chiroptical spectroscopy applications and reported successful outcomes. In the context of continuously increasing applications of chiroptical spectroscopy for chiral molecular structure determination, a review of recent developments and applications for chiral drugs is presented in this manuscript.

## 1. Introduction

Life on planet Earth owes its existence to chirality or handedness. Chiral molecules are those that possess handedness, i.e., in simple terms, they are either left handed or right handed. Only one type of handedness is preferred by the biological molecules that control the life machinery. L-amino acids and D-sugars, are the chiral constituents of proteins and nucleic acids that make up biological systems. As a consequence of the preferential dominance of chirality, drugs that are developed for the treatment of human diseases or upkeep of human health, have to possess the correct handedness. Determination of the handedness of chiral molecules, also referred to as the determination of absolute configuration (AC), has become an important research area. Chiroptical Spectroscopy [1,2] is becoming an invaluable tool for this purpose.

Historically the AC determination was accomplished using X-ray crystallography, nuclear magnetic resonance (NMR) or stereoselective synthetic schemes. These three methods require, respectively, good quality crystals, diastereomeric complexes and labor-intensive chemical synthetic procedures. These disadvantages are avoided in chiroptical spectroscopic applications, where investigations on native samples in solution phase (or vapor phase in favorable situations) can be directly undertaken.

Optical rotation (OR) in the visible spectral region is one chiroptical property that elicited interest in the early days [3]. When normalized with concentration (in g·cc^−1^) and pathlength (in dm), the resulting quantity is referred to as Specific rotation (SR), or specific optical rotation (SOR) as it is called in the recent literature. SOR is normally considered to be a molecular property that is independent of concentration, although exceptions are known [4]. In the older literature, attempts were made to correlate SOR with AC, mostly via empirical models, but they did not survive the test of time. Circular dichroism (CD) associated with electronic transitions, referred to as electronic CD (ECD), is another chiroptical property that was popularized among synthetic organic chemists by Nakanishi, Harada, Berova, and their coworkers, via the use of an exciton chirality model [5,6]. The scope of this approach is limited to molecules that possess two interacting electronic chromophores that absorb light in the visible spectral regions. A large array of chiral molecules, that do not contain electronic chromophores absorbing visible light, cannot be studied using this method. This limitation was overcome by the development of two new methods that measure optical activity associated with vibrational transitions, which do not depend on the absence or presence of electronic chromophores. One method utilizes the CD associated vibrational transitions, referred to as vibrational CD (VCD) [7] and the other utilizes the differential vibrational Raman scattering referred to as vibrational Raman optical activity (VROA) [8,9]. These two vibrational transition-based methods are applicable for all chiral molecules. The four methods, namely, SOR, ECD, VCD and VROA, are collectively categorized as chiroptical spectroscopic methods. The advances in chiroptical spectroscopic instrumentation and the availability of commercial instruments allowed many new researchers to undertake these measurements.

The experimental data obtained with any or all four of the chiroptical methods mentioned in the previous paragraph, however, cannot by themselves directly reveal the ACs of chiral molecules. These experimental data must be analyzed with theoretical predictions of corresponding chiroptical properties to infer the ACs.

The theoretical methods used for predicting chiroptical properties can range from approximate models to semi empirical methods and to modern quantum chemical (QC) methods. The predictions resulting from approximate models and semi empirical methods are not reliable enough for determining the ACs. Due to the revolutionary developments that took place in QC theories [10,11,12], theoretical predictions resulting from the QC calculations are providing high enough confidence to reliably analyze the experimental data. The availability of software to undertake the QC calculations, and of fast computer processors to expedite the calculations has made the applications of chiroptical spectroscopic methods routine in recent years.

The current practice of determining the ACs of chiral molecules involves experimental measurement of chiroptical properties for the chemical substance of interest and undertaking QC calculations for possible structures of the molecules making up that substance. These two steps are followed by comparative analysis of the experimental and predicted chiroptical spectra.

## 2. Chiroptical Spectroscopic Tools

In three of the following sub-sections, the instrumentation needed for experimental measurements and software used for QC predictions and spectral comparison analysis are summarized, to facilitate the introduction to newcomers. The fourth subsection illustrates a detailed procedure for AC determination.

### 2.1. Instrumentation

The instrumentation for optical rotation measurements for liquid samples is well established and commercial instruments are available (from Jasco Inc., Easton, MD, USA and Rudolph Research Analytical Inc., Hackettstown, NJ, USA). These instruments, which cost under $20,000, provide a measurement accuracy of 2 mdeg which is sufficient for most typical cases. Commercial laser polarimeters (from PDR Chiral Inc., Lake Park, FL, USA), which cost about $40,000, provide a measurement accuracy of 20 μdeg, and are mostly used with chromatography applications. Optical rotation measurements for gas phase samples can be undertaken using specialized instruments that use cavity ring down methods [13,14,15]. However, these instruments are quite expensive and are currently limited to academic laboratories. The instrumentation for ECD measurements for liquid samples is well established and commercial instruments are available (from Jasco Inc., Olis Inc., Bogart, GA, USA; Applied Photophyscis Inc., Leatherhead, Surrey, UK). These instruments cost ~$150,000. The instrumentation for VCD measurements for liquid samples is well established and commercial instruments (from Jasco Inc., Bruker Inc., Billerica MA, USA; BioTools Inc., Jupiter, FL, USA) cost under ~$200,000. The instrumentation for VROA measurements for liquid samples is also well established and currently there is only one commercial instrument (from BioTools Inc.), which costs ~$275,000.

### 2.2. QC Software

QC calculations of chiroptical properties can be undertaken on a variety of computer platforms, ranging from desktop computers to supercomputing clusters, using commercially available as well as freeware software. Freeware packages include DALTON [16], Psi4 [17] and NWChem [18], while popular commercial software packages include Gaussian [19] and ADF [20].

### 2.3. Spectral Comaprison Software

Spectral comparison involves comparing the digital experimental spectra with corresponding predicted spectra using spectral overlap integral methods [21,22]. A general purpose freeware program, *CDSpecTech*, has been developed in the author’s laboratory, to analyze the ECD, VCD and/or VROA spectra, corresponding absorption/Raman spectra and also their ratio spectra [23]. *SpecDis*, a program restricted to the analysis of ECD and corresponding absorption spectra, is also a freeware program [24]. *CompareVOA* (from BioTools Inc.) is a commercial program for analyzing the VCD and corresponding absorption spectra [25].

### 2.4. A Step-by-Step Guide for AC Determination

**Figure 1:**
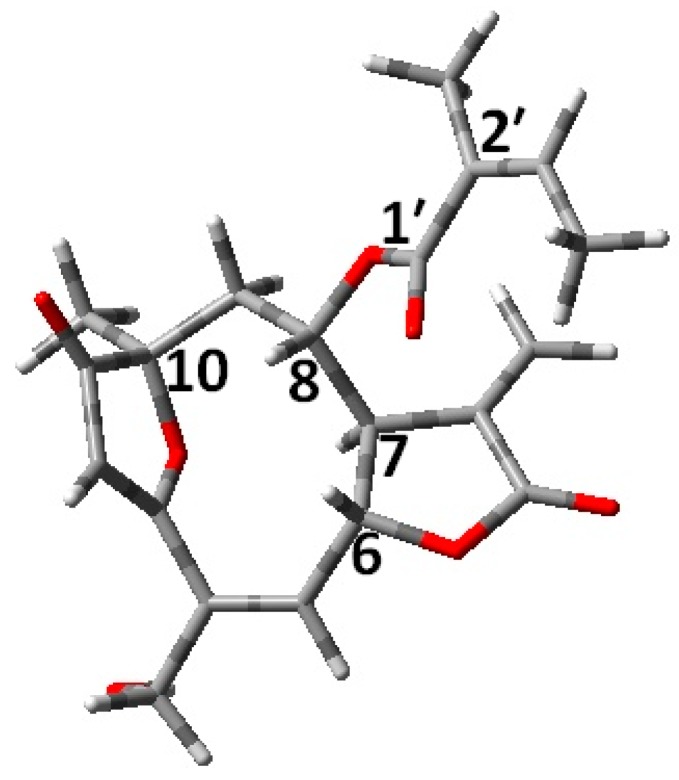
The (6*R*,7*R*,8*S*,10*R*,2’*Z*) structure of centratherin [26].

Centratherin (see Figure 1), a natural product with anti-inflammatory and anti-microbial properties, will be used in this article as an example to lead the reader through different steps involved in the AC determination [26].

The experimental chiroptical spectral data are obtained for the sample of interest dissolved in an appropriate solvent. If the solvent used does not participate in solute-solvent intermolecular hydrogen bonds, then corresponding QC calculations can be handled, relatively easily, with implicit solvation models. If intermolecular hydrogen bonding between solvent and solute is involved, then corresponding QC calculations need to incorporate explicit solvent molecules to effectively incorporate solvent effects into the calculations. The use of solvents that do not participate in intermolecular hydrogen bonding are to be favored, unless the goal is to unravel the intermolecular hydrogen bonding itself. Nevertheless, experimental measurements in different solvents at different concentrations are useful to assess the influence of solvents and concentrations.

**Figure 2:**
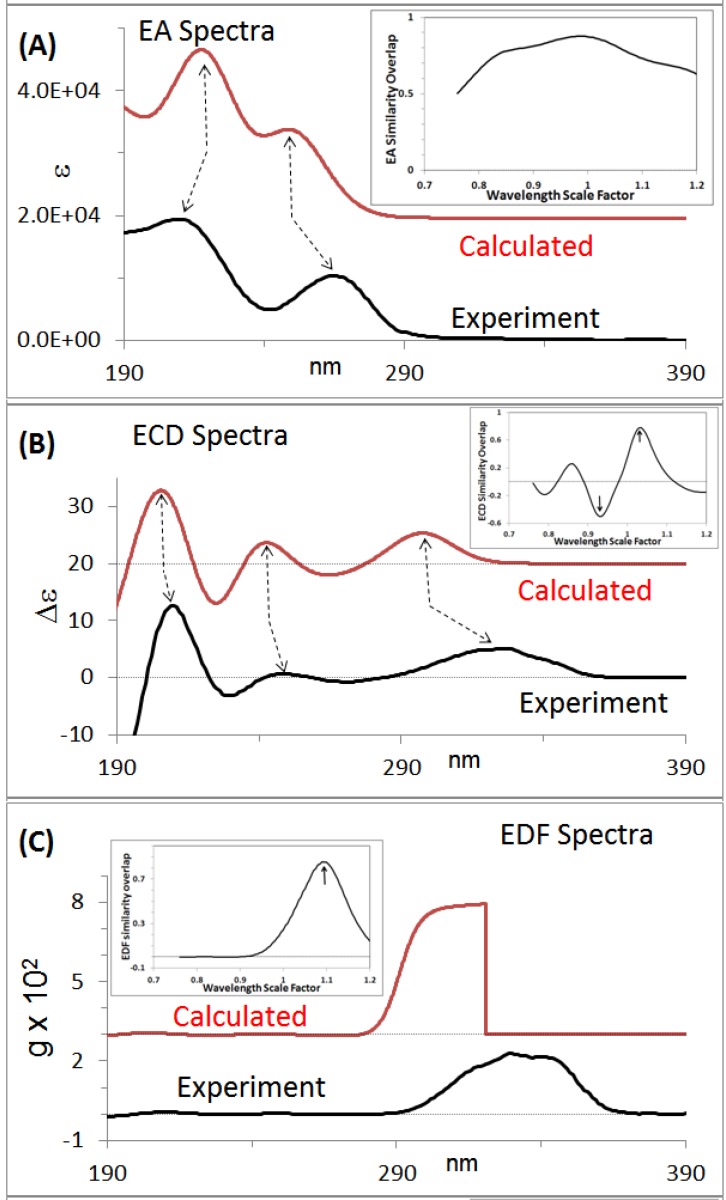
(**A**) Experimental and predicted electronic absorption spectra. Inset shows the similarity overlap plot as a function of wavelength scale factor; (**B**) experimental and predicted ECD spectra. Inset shows the similarity overlap plot as a function of wavelength scale factor; (**C**) experimental and predicted EDF spectra. Inset shows the similarity overlap plot as a function of wavelength scale factor. The experimental spectra are for (−)_589_-centratherin and predicted spectra are for (6*R*,7*R*,8*S*,10*R*,2’*Z*) structure. Data taken from Reference [26].

**Figure 3:**
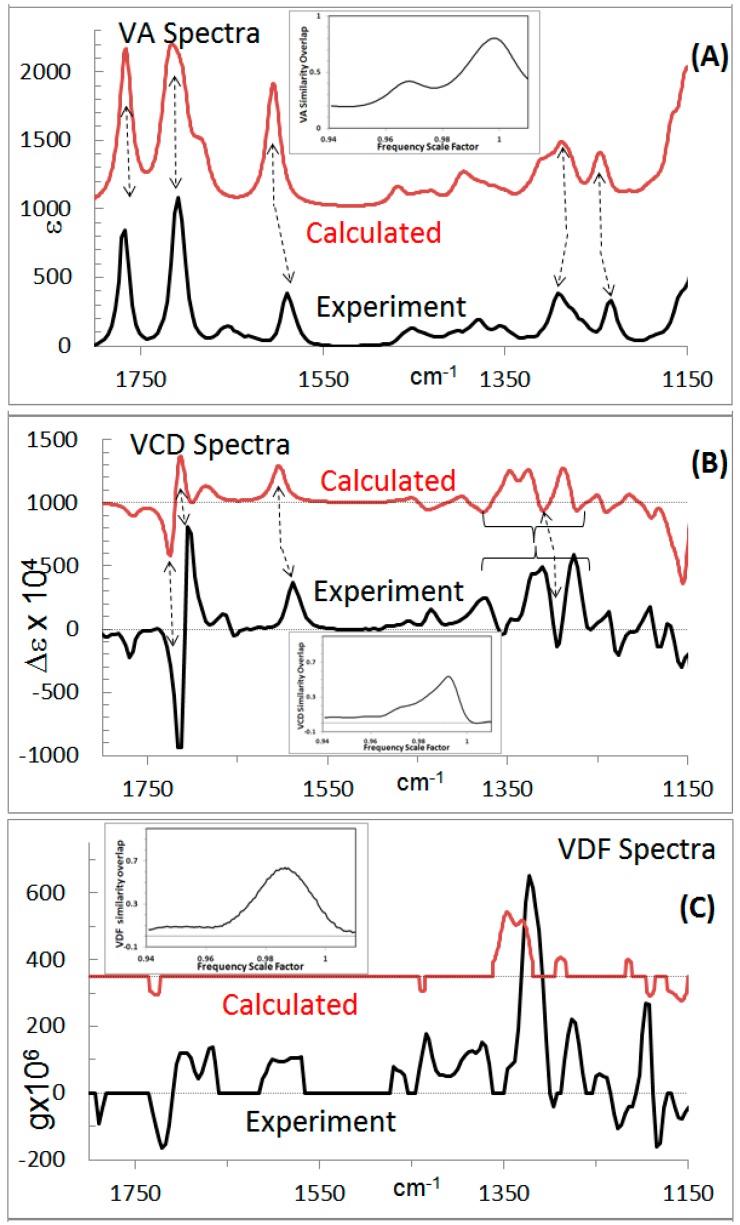
(**A**) Experimental and predicted vibrational absorption spectra. Inset shows the similarity overlap plot as a function of wavenumber scale factor; (**B**) experimental and predicted VCD spectra. Inset shows the similarity overlap plot as a function of wavenumber scale factor; (**C**) experimental and predicted VDF spectra. Inset shows the similarity overlap plot as a function of wavenumber scale factor. The experimental spectra are for (−)_589_-centratherin and predicted spectra are for (6*R*,7*R*,8*S*,10*R*,2’*Z*) structure. Data taken from Reference [26].

The experimental ECD and VCD spectra and corresponding absorption spectra for centratherin measured in acetonitrile solvent [26] are shown in Figure 2 and Figure 3, respectively. The experimental ROA spectra could not be measured for centratherin due to fluorescence issues and lack of the availability of enough sample. The experimentally measured SOR for centratherin [26] at 589 nm is −12 deg·cc·g^−1^·dm^−1^. Since the sample used for experimental measurements has negative SOR at 589 nm, this sample will be labeled as (−)_589_-centratherin.

Once a reliable set of experimental data has been gathered, QC calculations of chiroptical properties are initiated. The basic requirement for theoretical predictions of chiroptical properties is that the correct chemical structure of the compound of interest is known. Here chemical structure is meant to represent the chemical composition (atomic constituents) and the atomic connectivities (which atom is connected to which). Given that information, the three dimensional arrangement of atoms in a chiral molecule is the question that will be addressed by chiroptical spectroscopy. The three dimensional arrangement of atoms in a chiral molecule is defined by the ACs at individual chiral centers and dihedral conformations. Quite often, the relative ACs of the chiral centers may have been determined in the literature. If relative ACs are known with definite certainty, then theoretical predictions can be undertaken for that one diasteromer and conformational analysis conducted to predict the chiroptical properties. For situations where there is no prior information on the ACs of individual chiral centers, one would need to consider all possible diastereomers (and dihedral conformations associated with each diastereomer) and predict the chiroptical properties for all those possibilities. For a compound possessing “n” sources of chirality there will be 2^n^ diasteromers. Here, a source represents many of the types of chirality (for example, central chirality, axial chirality, etc.). One needs to consider 2^n−1^ diastereomers for theoretical predictions, because one-half of the 2^n^ diastereomeric structures are mirror images of the other half and their chiroptical properties would be opposite of the other half. For compounds possessing multiple sources of chirality, this situation can turn into a computational nightmare. More often than not, ACs for some of the chiral centers may have already been determined by other means (such as NOEs from NMR spectra), which will permit fixing the ACs at certain centers in the molecule, thereby reducing the computational demand.

The relative AC of centratherin (see Figure 1) is known from the literature as (6*R*,7*R*,8*S*,10*R*,2’*Z*), and therefore this disatereomer was used for the theoretical calculations [26].

The first step in the process for theoretical predictions is to build a molecular model. This involves pre-defining the chirality at individual chiral centers (and axial chirality, if one is involved) of the molecule of interest. There are several molecular visualization programs that can be used to build the molecular models. A popular freeware program is Avogadro [27]. Then a conformational search is carried out for the starting structure. A variety of commercial conformational search programs (Spartan [28], Conflex [29], Hyperchem [30], Macromodel [31]) are available for this purpose. It is important to make sure that all of the conformations found belong to the same diastereomer. If some conformers are found to have altered ACs then they need to be appropriately modified before further processing. The conformations determined with conformational search programs, within 20 kcal/mol of energy, need to be further refined using QC programs via geometry optimization options. A good compromise for the QC level of theory to be used here is B3LYP functional and 6-31G* basis set. The energies of optimized geometries at this level are compared and the lowest energy conformers within a certain energy range are retained for further geometry optimization at a higher level of theory. It is important to verify that the optimized conformers are at the minimum of potential energy, by performing vibrational frequency calculations and ensuring that there are no imaginary vibrational frequencies.

For the (6*R*,7*R*,8*S*,10*R*,2’*Z*) structure, sixteen lowest energy conformations, within 3 kcal/mol range at B3LYP/6-31G* level were reoptimized at B3LYP/aug-cc-pVDZ level of theory [26].

Then, chiroptical properties are calculated for all of these selected conformers with one particular AC. For the OR phenomenon, the predicted chiroptical property is the SOR at specific wavelengths. The chiroptical properties predicted for the CD phenomenon are the rotational strengths for each of the transitions. For the corresponding absorption phenomenon, the predicted properties are dipole strengths for each of the transitions. For the ROA phenomena, the properties predicted include normal coordinate derivatives of three different polarizability tensors (namely, electric dipole-electric dipole (EDED), electric dipole-magnetic dipole (EDMD) and electric dipole-electric quadrupole (EDEQ)) for each of the vibrational transitions. The anisotropies of these tensors are combined in an appropriate form to derive the activity associated with the ROA phenomena [9,32,33]. The anisotropy of EDED polarizability derivative tensor determines the activity associated with Raman spectra.

Rotational strengths in the case of CD phenomenon, and activities in the case of ROA phenomenon, are merely the signed numbers that represent integrated band intensities in the CD or ROA spectra. Dipole strengths in the case of absorption associated with CD, or Raman activities in the case of Raman associated with ROA, are positive numbers that represent integrated absorption band intensities in absorption or Raman spectra. They all can only be compared with corresponding integrated band intensities in the experimentally measured spectra. The task of integrating the band areas in the experimentally measured spectra is laborious, and may not provide easy comparison to the predicted quantities when multiple transitions overlap. Therefore, the opposite procedure is used by converting the predicted integrated band intensities into simulated predicted spectra, which can be compared with experimentally observed spectra. Simulation of theoretical spectra involves the assumptions of a band profile (Gaussian, Lorentzian, etc.) and a bandwidth for each of the transitions. For convenience, all transitions are assumed to have the same band profile and one chosen bandwidth. All these simulations can be conveniently undertaken using the spectral comparison software [23]. Since SOR is a cumulative effect from all electronic transitions, there is no simulation involved for theoretical SOR predictions.

For the (6*R*,7*R*,8*S*,10*R*,2’*Z*) structure, sixteen lowest optimized energy conformations were used for ECD and ORD calculations at CAM-B3LYP/aug-cc-pVDZ level and for VCD at B3LYP/aug-cc-pVDZ level of theory.

The calculated chiroptical properties for individual conformers (all with the same predefined AC) are to be weighted with their percent populations, referred to as Boltzmann populations or weights. These Boltzmann weights can be determined from the electronic energies or Gibbs energies obtained in the QC calculations. The Boltzmann weighted predicted ECD and VCD spectra for (6*R*,7*R*,8*S*,10*R*,2’*Z*) diastereomer are shown in Figure 2B and Figure 3B, while the corresponding absorption spectra are shown in Figure 2A and Figure 3A. The predicted population weighted SOR, for (6*R*,7*R*,8*S*,10*R*,2’*Z*) structure, is −44 deg·cc·g^−1^·dm^−1^ at 589 nm.

*Visual analysis of chiroptical spectra*: At the simplest level of spectral comparison analysis, the experimental and predicted chiroptical spectra are visually compared to assess the agreement between them. To facilitate the visual comparison, lines are drawn correlating the bands observed in the experimental spectra with those in the calculated spectra. In the case of ORD, SOR values measured at discrete wavelengths are compared to those obtained in the calculations (keeping in mind that the calculated wavelengths may have been shifted from the experimental wavelengths). From Figure 2 and Figure 3, it can be seen that the experimental data for the enantiomer with negative SOR at 589 nm matches that predicted for (6*R*,7*R*,8*S*,10*R*,2’*Z*) diastereomer. Therefore, the AC of centratherin can be assigned [26] as (−)_589_-(6*R*,7*R*,8*S*,10*R*,2’*Z*).

Most of the reported chiroptical spectral analyses for chiral drugs were accomplished via visual spectral analyses. A table summarizing the chiral drugs whose ACs were investigated [34,35,36,37,38,39,40,41,42,43,44,45,46,47,48,49,50,51,52,53,54,55,56,57,58,59,60,61,62,63,64,65,66,67,68,69,70,71,72,73,74,75,76,77,78,79,80,81,82,83,84,85,86,87,88,89,90] using chiroptical spectroscopic methods are summarized in Table 1. Occasional reviews have also highlighted some of these chiral drugs [42,65,91,92]. There are several natural products with promising medicinal values where ACs were investigated using chiroptical spectroscopy, but it will be unwieldy to review all of them here.

### 2.5. Spectral Overlap Analysis

Visual spectral analysis does not provide any quantitative measure of agreement between experimental and predicted spectra and may introduce inadvertent user bias. To provide a quantitative measure of agreement, and eliminate the unintended bias, the digital experimental and predicted spectral data are used to calculate the normalized overlap integral between them. If the normalized overlap integral for signed chiroptical spectral data is 1, then the experimental data of the enantiomer used for experimental measurements are reproduced exactly by the AC used for calculations. On the other hand, if the normalized overlap integral for signed chiroptical spectral data is −1, then, the experimental data of the enantiomer used for experimental measurements would be reproduced exactly by the AC, which is opposite to that used for calculations. The normalized overlap integrals for the absorption and Raman spectra, which are between 0 and 1, do not reflect the AC. Nevertheless, it is important to verify that the predicted absorption or Raman spectrum also reproduces the corresponding experimental spectrum (vide infra). Similarity overlap plots for absorption and CD spectra are shown as insets in Figure 2A,B and Figure 3A,B. The ECD spectral overlap analysis (see inset in Figure 2B) yields a ECD spectral overlap of 0.78 for (−)_589_-(6*R*,7*R*,8*S*,10*R*,2’*Z*) at a wavelength scale factor of 1.03 and also a overlap of −0.5 at a wavelength scale factor of 0.93. The later value indicates the level of agreement for the opposite enantiomer. This information could not have been obtained from the visual spectral analysis. Even though the overlap for (−)_589_-(6*R*,7*R*,8*S*,10*R*,2’*Z*) is higher and that for the opposite enantiomer is lower, one should not be overconfident in assigning the AC from ECD spectral overlap alone (vide infra). The VCD spectral overlap analysis (see inset in Figure 3B) yields a overlap of 0.53 at a frequency scale factor of 0.99 for (−)_589_-(6*R*,7*R*,8*S*,10*R*,2’*Z*) and none with opposite signs. Thus any possible assignment of opposite AC is ruled out from the VCD spectral analysis. Comparison of ORD data also supports (−)_589_-(6*R*,7*R*,8*S*,10*R*,2’*Z*) assignment [26]. Thus, different chiroptical spectra provide complimentary information that that should be made use of. The spectral overlap analyses discussed in this paragraph have been reported for only a limited number of chiral drugs summarized in Table 1.

*Advanced Spectral analysis*: When chiroptical spectra are analyzed, a concomitant analysis for accompanying absorption and Raman spectra should also be provided. This is because some of the fundamental quantities involved are common to both of them. However, undertaking separate analyses for them represents an incomplete enquiry. A complete analysis includes comparison of ratio spectra, i.e., ratio of CD to absorption spectra and ratio of ROA to Raman spectra. The former is called dimensionless dissymmetry factor (DF), while the latter is called dimensionless circular intensity difference (CID). The ratio of ECD spectrum to corresponding absorption spectrum is called electronic dissymmetry factor (EDF) spectrum and the ratio of VCD spectrum to corresponding absorption spectrum is called vibrational dissymmetry factor (VDF) spectrum. The EDF and VDF spectra for centratherin are shown in Figure 2C and Figure 3C. The EDF spectral overlap analysis (see inset to Figure 2C) for (−)_589_-(6*R*,7*R*,8*S*,10*R*,2’*Z*) yields a value of 0.85 and no negative values. Note that, in the previous paragraph, we stated that ECD spectra indicated a possibility for the opposite AC assignment, but EDF spectra now rule out that possibility. This example clearly shows the usefulness of EDF spectra. The VCD spectral overlap analysis (see inset to Figure 3C) for (−)_589_-(6*R*,7*R*,8*S*,10*R*,2’*Z*) yields a value of 0.62 and no negative values. The overlap values for EDF and VDF are greater than the corresponding values for ECD and VCD. Thus the advanced spectral analysis also suggests the AC of centratherin as (−)_589_-(6*R*,7*R*,8*S*,10*R*,2’*Z*).

The DF spectral analysis provides more insight than CD spectra alone. Furthermore, as literature studies indicate [69,93], the spectral overlap analysis for DF can be particularly useful when a distinction needs to be made out of several possible diastereomers. In addition, unexpected new information may ensue [94] from DF spectral analyses.

Since DF spectral analyses is a new concept, these analyses have not been yet been generally adopted. However, future investigations are recommended to incorporate the DF analyses, as there is more to gain and nothing to lose in such analyses.

Finally, it should be noted that for molecules with several chiral centers with unknown ACs, a unique solution results when only one diastereomeric structure provides the best match between experimental and predicted chiroptical property. But if multiple diastereomeric structures provide similar matches to the experiment, which is a common occurrence, then a given chiroptical property could not have provided the unique solution for AC. This later situation can be avoided if more than one chiroptical property is simultaneously analyzed for the compound of interest. This is because even if multiple diastereomers may possess the same chiroptical property in a given chiroptical method it is unlikely that they will have the same chiroptical property in another chiroptical method.

## 3. Conclusions

The pharmaceutical industry has widely adopted the chiroptical spectroscopic methods for establishing the ACs of chiral drugs that they are developing. There are some improvements that can be incorporated in to the methods that are being routinely used. The analysis of DF and CID spectra can improve the reliability of the AC assignments, help discriminate among multiple diastereomers and provide better insight. It is pragmatic, and generally important, to investigate more than one chiroptical property for determining the ACs of chiral molecules.

## Figures and Tables

**Table 1:** Chiral molecules with pharmaceutical applications investigated for their absolute configurations.

Molecule	Methods Used	Practical Use	References
(1*S*,2*S*,3*R*)-1-acetoxymethyl-2,3,4,4-tetra-methylcyclopentane	VCD	pheromone	[34]
Aeroplysinin-1	VCD, ROA	treatment of antiangiogenic disease	[35]
(*S*)-alaptide	ECD, ORD	veterinary ointment	[90]
(*S*)-alternarlactam	VCD	fungal cytotoxin	[36]
(*S*)-AM3189	VCD, OR	diabetes treatment	[37]
(1*S*,4*S*)-amino-aza-benzimidazolone	VCD	positive allosteric modulators	[38]
(*P*)-9-amino-2-cyclopropyl-5-(-2-fluoro-6-methoxyphenyl)-2,3-dihydro-pyrrolo[3,4-*b*]quinolin-1-one	VCD	atropisomers of GABA modulator	[39]
(*S*,*R*,*R*)-Aprepitant	VCD	neurokinin 1 (NK1) receptor antagonist,	[40]
(1*R*,5*S*)-1-azabicyclo[3.1.0]hexane	VCD	principle structural fragment of antitumor antibiotics azinomycins A and B	[41]
(*S*)-AZD624	VCD	NK3 antagonist	[42]
(*S*)-AZD6765	VCD	NMDA antagonist	[42]
(*R*,*S*) and (*S*,*S*)-Bedaquiline Analogs	ECD	Antituberculosis Agents	[43]
(*S*)-benzodiazepine	VCD	Active component of vasopressin receptor antagonist	[44]
(*S*) and (*R*)-2-(3,5-bis(trifluoromethyl)phenyl)-*N*-(4-(2-methylpyridin-4-yl)phenyl)propanamide	VCD, OR	γ-Secretase modulators	[45]
(2*S*,5*R*,6*S*)-2-Benzyl-5,6-bis(4-bromophenyl)-4-methylmor-pholin-3-one	VCD, OR	MDM2 inhibitor	[46]
(*R*) 8-(4-bromophenyl)-8-ethoxy-8*H*-[1,4]thiazino[3,4-*c*][1,2,4]-oxadiazol-3-one	ECD, ORD, VCD	Calcium Channel antagonist	[47,48]
(*S*) and (*R*)-(4-bromophenyl)(imino)(trifluoromethyl)-λ^6^-sulfanone	VCD	synthesis of Glucokinase regulatory protein disruptor	[49]
(*S*) and (*R*)-(6-bromopyridin-3-yl)(methyl)(trifluoromethyl)-λ^4^-sulfanol	VCD	synthesis of Glucokinase regulatory protein disruptor	[49]
(1*R*,6*S*,9*R*)-Buagafuran	ECD	antianxiety	[50]
(6*R*,7*S*)-chromenotriazolopyrimidine	VCD	MDM2-p53 inhibitor	[51]
(2*R*,3*R*) and (2*S*,3*S*)-2-chlorophenylglycidol	VCD, OR, ECD	functionality that can yield intermediates for the synthesis of chiral drugs	[52]
(2*R*,3*R*) and (2*S*,3*S*)-3-chlorophenylglycidol	VCD, OR, ECD	functionality that can yield intermediates for the synthesis of chiral drugs	[52]
(2*R*,3*R*) and (2*S*,3*S*)-4-chlorophenylglycidol	VCD,OR,ECD	functionality that can yield intermediates for the synthesis of chiral drugs	[52]
(*S*)-1-[(4-chlorophenyl)sulfonyl-2-(2-thienyl)pyrrolidine	VCD	Calcium Channel antagonist	[53]
(*S*)-2-(2-chloro-4-pyridinyl)-1,1,1-trifluoro-2-propanol	VCD,OR	synthesis of Glucokinase regulatory protein disruptor	[54]
(1*S*,2*S*)-Cipralisant (GT-2331)	VCD	H_3_ antagonist	[55]
(*S*)-3-cyanomethyl-3-hydroxyindole	VCD, ECD	antifungal agent	[56]
(*S*)-desflurane	VCD, OR	anesthetic	
(*R*)-dichloroprop	VCD	herbicide	[57,58,59]
(*S*)-dioxybrassinin	VCD, ECD	antifungal agent	[56]
(*S*)-Efavirenz	VCD	antiretroviral	[40]
(*S*)-enflurane	VCD, OR	anesthetic	[61]
(1*S*,2*R*)-ephedrine	VCD, ROA	antiinflammatory	[62,63]
(*S*,*S*)-Ethyl 6’-hydroxy-2’,3’-dihydrospiro[cyclopropane-1,1’-indene]-2-carboxylate	VCD, OR	Head group in Potent GPR40 Full Agonists	[64]
(*S*)-etodolac	VCD	antiinflammtory	[65]
(1*S*,2*R*,3*S*)-Ezetimibe	VCD	to treat high cholesterol	[40]
(*R*,*R*)-Filorexant	VCD	Orexin Receptor Antagonist	[40]
(*S*)-Flecainide	VCD	antiarrhythmic	[60]
(2*R*,3*R*) and (2*S*,3*S*)-2-fluorophenylglycidol	VCD,OR,ECD	functionality that can yield intermediates for the synthesis of chiral drugs	[52]
(2*R*,3*R*) and (2*S*,3*S*)-3-fluorophenylglycidol	VCD, OR, ECD	functionality that can yield intermediates for the synthesis of chiral drugs	[52]
(2*R*,3*R*) and (2*S*,3*S*)-4-fluorophenylglycidol	VCD, OR, ECD	functionality that can yield intermediates for the synthesis of chiral drugs	[52]
(2*R*,3*R*) and (2*S*,3*S*)-4-(4-fluorophenyl)-3-hydroxymethyl-1-methylpiperidine	VCD	precursor to paroxetine and femoxetine	[52]
(1*R*,5*S*)-Frontalin	VCD	pheromone	[66]
(*P*)-Gossypol	VCD	Antineoplastic agent	[67]
(*R*)-4-hydroxywarfarin	ECD	anticoagulant	[68]
(*R*)-6-hydroxywarfarin	ECD	anticoagulant	[68]
(*R*)-7-hydroxywarfarin	ECD	anticoagulant	[68]
(*R*)-8-hydroxywarfarin	ECD	anticoagulant	[68]
(*S*)-Ibuprofen	VCD	antiinflammatory	[40,70]
(7*R*,8*R*,10*S*)-inuloxin A	ECD, ORD, VCD	phytotxin	[71]
(*S*)-Isoflurane	VCD, OR, ROA	anesthetic	[72,73]
(2*R*,4*S*)-itraconazole	VCD	antifungal agent	[74]
(2*R*,4*S*)-ketoconazole	VCD	antifungal agent	[74]
(*R*)-Laropiprant	VCD	prostaglandin D2 receptor 1 antagonist	[40]
(*R*)-malathion	VCD	pesticide	[75]
(6*S*,10b*R*)-McN 5652-X	VCD	transport of seratonin in brain	[76]
(*R*)-mecoprop	VCD	herbicide	[57,58,59]
(2*R*,3*R*) and (2*S*,3*S*)-3-methoxyphenylglycidol	VCD, OR, ECD	functionality that can yield intermediates for the synthesis of chiral drugs	[52]
(*S*)-miconazole	VCD	antifungal agent	[74]
(*R*)-mirtazapine	VCD	antidepressant	[77]
(*R*)-*N*-(Pyridin-3-yl)-1-(4-(trifluoromethyl)phenyl)-3,4-dihy-droisoquinoline-2(1*H*)-carboxamide	OR	TRPM8 antagonists	[78]
(*S*)-Naproxen	VCD	antiinflammatory	[60]
(*S*)-*N*-Cyclopropyl-4-methyl-3-[1-(2,2,2-trifluoro-1-methylethoxy)phthalazin-6-yl]benzamide	VCD	p38 MAP Kinase Inhibitor	[79]
(1*S*,2*R*)-*N*-methylephedrine	VCD	antiinflammatory	[62]
(1*S*,2*S*)-*N*-methylpseudoephedrine	VCD	antiinflammatory	[62]
(1*S*,2*R*)-norephedrine	VCD, ROA	antiinflammatory	[62,63]
(1*S*,2*S*)-norpseudoephedrine,	VCD, ROA	antiinflammatory	[62,63]
(*R*,*R*)-Otamixaban	VCD	fXa inhibitor	[80]
(*S*)-oxadiazol-3-one	VCD	calcium channel blocker	[48]
(4*S*,5*R*,6*R*)-Oxysporone	ORD, ECD, VCD	herbicides	[81]
paclitaxel	VCD	chemotherapy	[82]
(2*R*,3*R*) and (2*S*,3*S*)-Phenylglycidol	VCD, OR, ECD	functionality for the synthesis of chiral drugs	[52]
(3*S*,4a*R*,8*S*,8a*R*)-Phyllostin	ORD, ECD, VCD	herbicides	[81]
(*S*)-Propranolol	VCD	antiarrhythmic	[60]
(1*S*,2*S*)-pseudoephedrine	VCD, ROA	antiinflammatory	[62,63]
(*S*,*S*), (*R*,*S*), (*S*,*R*) and (*R*,*R*)-6-pyrimidin-2-yl-octahyro-pyrrolo[2,3-*c*] pyridine	VCD	core structure for synthesis of functionalized Sarain A	[83]
(1*S*,3*R*,4*S*,8*R*,9*S*)-Quinidine	VCD	antiarrhythmic	[60,84]
RAC-109	VCD	antiarrhythmic	[60]
(4a*R*,8*S*,8a*R*)-scytolide	VCD, ORD, ECD	herbicides	[81]
(1*S*,2*R*,3a*S*,4*S*,5*R*,7a*S*)-seiricardine A	ECD, ORD, VCD	phytotxin	[71]
(*R*,*S*,*R*,*S*,*S*,*R*,*R*)-Simvastatin	VCD	to lower cholesterol and triglycerides	[40]
(*S*) and (*R*)-Tetrahydroisoquinolines	VCD, OR	RPM8-Antagonists	[85]
(*R*)-Thalidomide	VCD	teratogen	[86]
(2*R*,3*R*) and (2*S*,3*S*)-2-trifluoromethylphenylglycidol	VCD, OR, ECD	functionality that can yield intermediates for the synthesis of chiral drugs	[52]
(2*R*,3*R*) and (2*S*,3*S*)-3-trifluoromethylphenylglycidol	VCD, OR, ECD	functionality that can yield intermediates for the synthesis of chiral drugs	[52]
(2*R*,3*R*) and (2*S*,3*S*)-4-trifluoromethylphenylglycidol	VCD, OR, ECD	functionality that can yield intermediates for the synthesis of chiral drugs	[52]
(*R*)-1,3,5-triphenyl-2-pyrazoline	ECD, VCD	anti depressant	[87]
Valinomycin	VCD, ROA	transport antibiotic	[88,89]
(*R*)-warfarin	ECD	anticoagulant	[68]
